# Synthesis, Characterization and Biological Activity of Dimethyltin Dicarboxylates Containing Germanium

**DOI:** 10.1155/MBD.2002.275

**Published:** 2002

**Authors:** M. A. Choudhary, M. Mazhar, S. Ali, X. Song, G. Eng

**Affiliations:** 1 Department of Chemistry University of Azad Jammu and Kashmir Muzaffarabad Muzaffarabad- 13100 Pakistan; 2 Department of Chemistry Quaid-i-Azam University Islamabad Pakistan; 3 Department of Chemistry and Physics University of the District of Columbia WashingtoD.C. USA

## Abstract

A series of diorganotin dicarboxylates of the general formula (CH_3_)_2_Sn(OCOCHR^3^CHR^2^GeR^1^)_2_ where R^1^=(C_6_H_5_)_3_, (P-CH_3_C_6_H_4_)3, N(CH_2_CH_2_O)_3_, R^2^=C_6_H_5_, H, CH_3_, P-CH_3_OC_6_H_4_, P-ClC_6_H_4_, P-CH_3_C_6_H_4_, R^3^=CH_3_ and H, have been synthesized by the reaction of dimethyltin oxide with germanium substituted propionic acid in 1:2 molar ratio in toluene. The H_2_O formed was removed azeotropically using a Dean and Stark apparatus. All the compounds have been characterized by IR, multinuclear (^1^H, ^13^C, ^119^Sn) NMR, mass and Mössbauer spectroscopies. All compounds were found to have potential activity against bacteria.


### SYNTHESIS, CHARACTERIZATION AND BIOLOGICAL ACTIVITY OF DIMETHYLTIN DICARBOXYLATES CONTAINING GERMANIUM

M. A. Choudhary[1], M. Mazhar*[2], S. Ali[2], X. Song[3] and G. Eng[3]

[1] Department of Chemistry, University of Azad Jammu and Kashmir, Muzaffarabad-13100 <azizch@yahoo.com>
[2] Department of Chemistry, Quaid-i-Azam University, Islamabad, Pakistan <Mazhar 42pk@yahoo.com>
[3] Department of Chemistry and Physics, University of the District of Columbia, WashingtoD.C., USA <geng@udc.edu>

**Abstract**

A series of diorganotin dicarboxylates of the general formula $(CH_3)_2Sn(OCOCHR^3CHR^2GeR^1)_2$ where $R^1 = (C_6H_5)_3$, $(p\text{-}CH_3C_6H_4)_3$, $N(CH_2CH_2O)_3$, $R^2 = C_6H_5$, H, $CH_3$, $p\text{-}CH_3OC_6H_4$, $p\text{-}ClC_6H_4$, $p\text{-}CH_3C_6H_4$, $R^3 = CH_3$ and H, have been synthesized by the reaction of dimethyltin oxide with germanium substituted propionic acid in 1:2 molar ratio in toluene. The $H_2O$ formed was removed azeotropically using a Dean and Stark apparatus. All the compounds have been characterized by IR, multinuclear ($^1H$, $^{13}C$, $^{119}Sn$) NMR, mass and Mössbauer spectroscopies. All compounds were found to have potential activity against bacteria.

### Introduction

Organotin chemistry has been the subject of much interest in recent years. The importance of this area of main group organometallic chemistry is in part a result of their various industrial and agricultural applications. In addition, they have been reported as having potential antitumor activities. These are well cited in the literature [1-5]. For example, organotin carboxylates have been reported by Gielen *et. al* to have promising activity against various antitumour cells [5]. Furthermore, Crowe *et. al* stated that the $R_2Sn^{2+}$ moiety is the active portion of the diorganotin, $R_2SnXY$, molecules. The function of the XY groups in these compounds is to transport the potentially active $R_2Sn^{2+}$ moiety to the site of action where it is released by hydrolysis [4].

There has been considerable interest in recent years in the chemistry of bioactive germanium compounds. The first organogermanium pharmaceutical propagermanium was launched in Japan in 1994. Its biological activity spectrum modules the protection against viruses, immunostimulation and hepatoprolation [ 6-10 ]. In the present work, we are reporting the synthesis of several germatranyl and triaryl germyl substituted propionic acids and their reactions with dimethyltin oxide. Compound I was found to be more active for *Bacillus cerus* and *Klebslella pneumoniae* than the reference drugs.

### EXPERIMENTAL

*Synthesis of Compounds*

Germanium-substituted propionic acids were prepared according to the literature [11] using Scheme 1. The germanium-substituted dimethyltin dipropionates were then synthesized by the condensation of dimethyltin oxide and germanium substituted propionic acids in a 1:2 molar ratio in toluene/ethanol (3:1). The general reaction is shown as follows:

$$Me_2SnO + 2HO_2CCHR^3CHR^2GeR^1 \longrightarrow Me_2Sn[OCOCHR^3CHR^2GeR^1]_2 + H_2O$$

The following is a typical procedure for the synthesis of the dimethyltin germanium substituted carboxylates: 0.005 Mole of dimethyltin oxide and 0.01 moles of appropriate germanium substituted propionic acids were suspended in a ethanol:toluene mixture (1:3) and refluxed for 10 hours. The water formed during the reaction was removed by a Dean and Stark apparatus. The solvent was removed under vacuum and the solid thus obtained was recrystallized from a chloroform:petroleum ether mixture (1:1). The yields and physical data are listed in Table 1.

*Spectra*

The infrared spectra were recorded as KBr discs on a Hitachi model 270-1117 spectrophotometer. PMR spectra were recorded in $CDCl_3$ on a Bruker SF 300 or SF 400 spectrometer using TMS as the internal reference. A Jeol FX90Q instrument using $Me_4Sn$ as the external reference was used to record the $^{119}Sn$ NMR. The mass spectral data were measured on a JMS-DX 300 mass spectrometer. The Mössbauer spectra were measured at 80K on a Ranger Model MS-900 Mössbauer spectrometer in the acceleration mode with a moving source geometry using a liquid nitrogen cryostat. The samples were mounted in Teflon holders. The source was 5 mCi

$Ca^{119m}SnO_3$, and the velocity was calibrated at ambient temperature using a composition of $BaSnO_3$ and Sn foil (splitting – 2.52 mm $s^{-1}$). The resultant spectra were analyzed by a least-square fit to Lorenzian shaped lines.

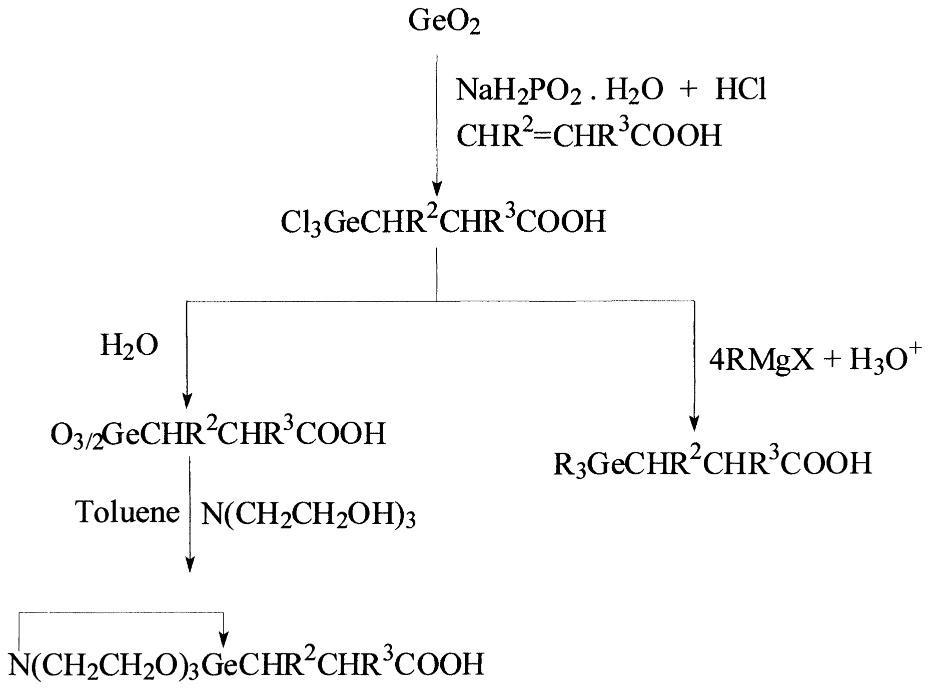

**Scheme 1.**

*Antibacterial activity*

The agar well diffusion technique was adopted for determining the antibacterial activity of the test compounds. Two mg/mL of the test solution were added to their respective wells. Other wells supplemented with DMSO and reference antibacterial drugs were used as negative and positive controls, respectively. The bacterial inocula (2-8 hours old) containing *ca.* $10^4$–$10^6$ colony forming units (CFU)/mL were then spread on the surface of the nutrient agar plates using a sterile cotton swab. The plates were incubated immediately at 37°C for 14-19 hours. After the incubation period, the zones of inhibition were calculated.

### Results and Discussion

*Infrared spectra*

The infrared spectra of the compounds have been recorded in the range of 4000-400 $cm^{-1}$ and the absorptions of interest are reported in Table 2. These absorptions include the OCO, Sn–C, Sn–O, Ge–O and Ge←N vibrations. The medium to weak bands in the region 430-470 $cm^{-1}$ are assigned to the Sn–O vibrations whereas absorptions in the region 500-630 $cm^{-1}$ indicates the presence of the Sn–C bonds [12,13]. The Ge–O bond absorbs in the region around 898 $cm^{-1}$ and the Ge←N coordination is found in the region of 680-695 $cm^{-1}$ in germatrane derivatives.

It has been reported that the shifting of the $\nu_{asym}$(COO) vibration to a lower frequency coupled with the shifting of the $\nu_{sym}$(COO) vibration to a higher frequency for the carboxylate group when compared to the ionic carboxylate values is indicative of a bidentate carboxylate group. For unidentate coordination of the carboxylate group the reverse is true [14]. Thus, the mode of coordination of the carboxylate group has been related to the magnitude of the separation (Δν) of the $\nu_{asym}$(COO) and $\nu_{sym}$(COO) vibrations [14]. In compounds (I-X), the Δν values in the solid are between 170-202 $cm^{-1}$. This range of Δν values is indicative of carboxylate groups that behave as a bidentate ligand.

The observation of both the Sn-C symmetric and asymmetric vibrations would indicate the C-Sn-C moiety is not linear. Based on these two observations the compounds are six-coordinated with non linear methyl groups in the solid state.

*Mössbauer spectra*

Indirect evidence for solid state structures of organotin compounds can also be derived from Mössbauer spectroscopy. In this context, the most useful parameter is the quadruple splitting (QS) for which a given range is associated with a particular coordination number and geometry at the tin atom. Mössbauer data for compounds II and III have QS values of 3.47 and 3.32 mm $s^{-1}$, respectively. This range of values suggests a *trans* $R_2Sn(O_2CR)_2$ structure with chelated carboxylate groups. Thus, the structures of the compounds in the solid state are hexacoordinated which is in agreement with the infrared results.

*NMR spectra*

The proton NMR spectral data of the complexes are given in Table 3. The observed resonances and patterns are in agreement with those expected for the titled compounds. The integrations of the spectra are in good agreement with the expected values for the protons in the complex molecules. Furthermore, the proton NMR spectra, in $CDCl_3$, of the cyclic skeleton of the simple germatranes consisted of two triplets ($A_2B_2$ spin system) at 2.23-2.83 ppm for the $NCH_2$ and 3.68-3.73 ppm for the $OCH_2$ protons. This pattern is the general feature for the atrane framework [15]. The relative values of the vicinal coupling constant are in the range of $^3JAB$ (6 Hz) and are consistent with the atrane framework [15].

In compounds (I-X), the methyl groups attached directly to tin atom absorbed in the range of 0.23-0.98 ppm and appears as a sharp singlet with $^2J(^{119}Sn-^1H)$ values ranging from 56 to 84 Hz. A particular advantage of methyltin derivatives is the ease with which proton spin-spin coupling constant can be determined. The coupling constants have been related to the hybridization state of the tin atom and has been reported to increase with an increase in coordination number [16]. The $^2J(^{119}Sn-^1H)$ values can be used to estimated the C-Sn-C bond angle using the equation $\theta = 0.0161\ |\ ^2J(^{119}Sn-^1H)\ |^2 - 1.32\ |\ ^2J(^{119}Sn-^1H)\ | + 133.4$ [17]. Using this criterion, the estimated bond angles for the compounds in solution range from 106° to 136°. This magnitude for the C-Sn-C bond angles has been reported in the literature for methyl compounds as being 5 or 6 coordinated [17].

The $^{13}C$ NMR spectral data are given in Table 4. The methyl carbon attached to tin atom absorbs in the range of 5 to 19 ppm. In the germatrane derivatives, the carbon atoms attached to the germanium atom through the $OCH_2$ and $NCH_2$ groups resonate at 56 and 51 ppm, respectively. The $^{13}C$ positions of the substituents on the phenyl rings are reported in parenthesis (Table 4). The $CH_2$ group attached to the COO group was observed to absorb at a lower field as compared to the CH group attached to the germanium atom (Table 4). Phenyl carbons absorb in the expected region and the carbonyl carbon of the COO group absorbed in the range of 170-180 ppm as was reported earlier [18-21]. The $^1J(^{119}Sn-^{13}C)$ coupling constant values ranges from 522 to 633 Hz. The recorded $^1J(^{119}Sn-^{13}C)$ coupling constant would suggest that these compounds are five or six-coordinated. For example, $Me_2\ Sn(OAc)_2$, a known hexacoordinated complex has a $^1J(^{119}Sn - ^{13}C)$ coupling constant of 660 Hz [17]. In addition, using equation, $|^1J(^{119}Sn-^{13}C)| = 11.4\theta - 875$ [17], the C-Sn-C bond angle can be estimated which ranges 122.5° to 133.28 which would support the suggestion that these compounds are five or six-coordinated as indicated by the observed $^1J(^{119}Sn-^{13}C)$ coupling constant and the estimated bond angle also in agreement with the bond angles obtained by the $^2J(^{119}Sn-^1H)$ coupling constants in the $^1H$ NMR studies.

Listed in Table 4 are also the $^{119}Sn$ NMR data. The $^{119}Sn$ chemical shifts cover a range of approximately 6500 ppm depending upon the coordination number [22]. It is generally accepted that the compounds with different geometries about the tin atom produce shifts in moderately well defined ranges. The range is between +200 to –60 ppm for four coordinated compounds and from –90 to –330 ppm for five coordinated systems and –125 to –515ppm for hexacoordinated compounds [23]. Since compounds II, III and VIII have $\delta$ values between –300 to –500ppm, it would indicate that these compounds are either five or six-coordinated while the other compounds are five-coordinated since their $\delta$ values lie in the range of –100 to –200ppm. These results are consistent with the $^1H$ and $^{13}C$ nmr results.

Table 1. Physical data and elemental analysis for $(CH_3)_2Sn(OCOCHR^3CHR^2GeR^1)_2$

| No | $R^1$ | $R^2$ | $R^3$ | M.p. (°C) | Yield (%) | Elemental analysis: Found (Calculated) | | |
|---|---|---|---|---|---|---|---|---|
| | | | | | | C% | H% | N% |
| I | $C_6H_5$ | $C_6H_5$ | H | 160-162 | 75 | 63.58 (63.88) | 4.80 (4.94) | - |
| II | $C_6H_5$ | H | H | 132-133 | 68 | 58.32 (58.67) | 4.65 (4.88) | - |
| III | $C_6H_5$ | H | $CH_3$ | 144-146 | 73 | 59.19 (59.49) | 4.98 (5.17) | - |
| IV | $C_6H_5$ | $CH_3$ | H | 98-100 | 66 | 59.19 (59.49) | 5.07 (5.17) | - |
| V | p-$CH_3C_6H_4$ | $C_6H_5$ | H | 196-198 | 60 | 65.15 (65.50) | 5.40 (5.63) | - |
| VI | p-$CH_3C_6H_4$ | p-$OCH_3C_6H_4$ | H | 216-218 | 60 | 63.98 (64.22) | 5.38 (5.68) | - |
| VII | $N(CH_2CH_2)_3N$ | p-$ClC_6H_4$ | H | 138-140 | 59 | 40.02 (40.38) | 4.45 (4.62) | 2.89 (2.94) |
| VIII | $N(CH_2CH_2)_3N$ | $C_6H_5$ | H | 148-150 | 74 | 43.39 (43.54) | 4.95 (5.21) | 3.07 (3.17) |
| IX | $N(CH_2CH_2)_3N$ | p-$CH_3C_6H_4$ | H | 140-142 | 60 | 44.65 (44.87) | 5.19 (5.49) | 2.99 (3.07) |
| X | $N(CH_2CH_2)_3N$ | $CH_3$ | H | 183.185 | 57 | 34.65 (34.83) | 5.46 (5.54) | 3.65 (3.69) |

Table 2. Selected infrared vibrations in $cm^{-1}$ for the $(CH_3)_2Sn(OCOCHR^3CHR^2GeR^1)_2$.

| No. | ν(COO)Asym. | ν(COO) Sym. | Δν | ν(Ge-O) | ν(Ge←-N) | ν(Sn-C) | ν(Sn-O) |
|---|---|---|---|---|---|---|---|
| I | 1596 | 1398 | 198 | — | — | 615, 519 | 457 |
| II | 1576 | 1374 | 202 | — | — | 614, 541 | 461 |
| III | 1570 | 1378 | 192 | — | — | 619, 560 | 456 |
| IV | 1562 | 1377 | 185 | — | — | 615, 545 | 459 |
| V | 1560 | 1390 | 170 | — | — | 638, 544 | 459 |
| VI | 1598 | 1396 | 202 | | — | 614, 529 | 490 |
| VII | 1561 | 1383 | 178 | 896, 796 | 692 | 614, 547 | 488 |
| VIII | 1559 | 1366 | 193 | 897, 790 | 695 | 618, 543 | 444 |
| IX | 1550 | 1364 | 186 | 898, 820 | 684 | 615, 531 | 424 |
| X | 1579 | 1377 | 202 | 893, 777 | 680 | 608, 534 | 442 |

*Mass spectra*

Main fragment ions observed in the mass spectra of compounds (I-X) are listed in Table 5. The germatranyl substituted compounds have a base peak at 220 which is due to the $N(CH_2CH_2O)_3Ge^+$ species while aryl substituted germanium compounds give base peaks fragments for $Ph_3Ge^+$ or $(CH_3C_6H_4)_3Ge^+$. However, the molecular ion peak was not found in any of the compounds. Also, the isotopic effects have been observed in all the fragmentation ions containing germanium and tin.

In the mass spectra of the germatrane compounds, the peak of highest intensity is at m/z 220 which corresponds to the germatranyl ion. This is a result of the cleavage of the germanium carbon bond in the parent ion. This behavior is analogous to that observed for 1-allylgermatrane and 1-fluorenyl germatrane [24] and is assumed to be a reflection of the relative strength of the germantrane skeleton.

In the mass spectral fragmentation of aryl-substituted germaniums $R_3Ge$ (where R = $C_6H_5$ and $CH_3C_6H_4$), the peak of highest intensity is found at 305 and 347, respectively. The successive loss of the aryl groups takes place as given below:

$$\underset{305}{(C_6H_5)_3Ge^+} \longrightarrow \underset{228}{(C_6H_5)_2Ge^+} \longrightarrow \underset{151}{(C_6H_5)Ge^+} \longrightarrow \underset{74}{Ge^+}$$

$$\underset{347}{(CH_3C_6H_5)_3Ge^+} \longrightarrow \underset{256}{(CH_3C_6H_5)_2Ge^+} \longrightarrow \underset{165}{CH_3C_6H_5Ge^+} \longrightarrow \underset{74}{Ge^+}$$

Table 3. $^{1}H$ NMR data of dimethyltin carboxylate, $(CH_3)_2Sn(OCOCHR^3CHR^2GeR^1)_2$

| No. | Methyl | $R^2$ | $CHR^2$ | $CHR^3$ | $R^1$ | $R^3$ |
|---|---|---|---|---|---|---|
| I | 0.92(s) [62.0] | 6.89-7.07(m) | 2.94(d) (4.7) | 3.73(t) (4.7) | 7.23-7.36(m) | - |
| II | 0.96(s) [64.0] | - | 1.81(t) (9.5) | 2.48(t) (9.0) | 7.32-7.48(m) | - |
| III | 0.88(s) [56.0] | - | 2.1(m) | 2.8(m) | 7.23-7.50(m) | 1.18 (d) (6.5) |
| IV | 0.98(s) [67.4] | 1.2(d) (6.2) | 2.39(m) | 2.73(m) | 7.23-7.50(m) | - |
| V | 0.23(s) [63.0] | 6.89-7.1(m) | 2.66(m) | 3.55(m) | 7.06-7.23(m) 2.31(s) | - |
| VI | 0.33(s) [83.5] | 6.65(d) (8) 6.82(d) (8.6) 3.71(s) | 2.88(m) | 3.50(m) (5.4) | 7.06-7.2(m) 2.3(s) | - |
| VII | 0.67(s) [76.0] | 7.15(d) 7.19(d) | 2.89(m) | 3.89(m) | 2.74(t) (5.5) 3.68(t) (5.4) | - |
| VIII | 0.42(s) [81.2] | 7.17-7.29(m) | 2.92(m) | 3.89(m) | 2.73(t) (5.3) 3.68(t) (5.5) | - |
| IX | 0.60(s) [80.0] | 6.97(d) (7.6) 7.21(d) (7.6) 2.23(s) | 2.92(m) | 3.85(m) | 2.73(t) (5.5) 3.68(t) (5.5) | - |
| X | 0.75(s) [80.0] | 1.29(d) (6.7) | 2.48(m) | 2.75(m) | 2.83(t) (5.7) 3.73(t) (5.7) | - |

[ ] = $^{2}J[^{119}Sn - ^{1}H]$, ( ) = $^{3}J(^{1}H, ^{1}H)$, s = singlet, d = doublet, t = triplet, m = multiple

Thus, the mass spectra data supports the proposed structures of the compounds. Furthermore, the absence of observing fragmentation ions larger than for the molecular ions indicate that the molecules are monomeric. This observation supports the earlier IR and Mössbauer results.

*Antibacterial Activity*

The bactericide study results using compounds I – V as the toxicant are given in Table 6. In general, most of the compounds had similar activity to the various bacteria as the two reference drugs, Amoxicillin and Ampicillin. However in a few cases, the activities of the compounds were slightly higher than those observed for the reference drugs. For example, compound I was found to be more active for *Bacillus cerus* and *Klebslella pneumoniae* than the reference drugs. All compounds have shown good activity against all pesto bacteria.

Table 4. $^{13}C$ and $^{119}Sn$ NMR data of the dimethyltin carboxylate, $(CH_3)_2Sn(OCOCHR^3CHR^2GeR^1)_2$.

| No. | I | II | III | IV | V | VI | VII | VIII | IX | X |
|---|---|---|---|---|---|---|---|---|---|---|
| SnMe | 6.3 [535] | 5.1 [522] | 6.3 [633] | 13.0[ 548] | 8.8 [568] | 3.9 [555] | 14.9 [539] | 19.5 [588] | 14.9 [540] | 17.6 [560] |
| $CHR^2$ | 31.8 | 9.1 | 20.4 | 18.4 | 30.2 | 36.9 | 37.1 | 24.0 | 37.1 | 23.6 |
| $CHR^3$ | 36.6 | 29.5 | 35.5 | 38.1 | 32.6 | 31.8 | 37.2 | 42.0 | 37.5 | 35.5 |
| C=O | 179.3 | 183.0 | 179.0 | 180.0 | 180.8 | 183.3 | 173.2 | 178.3 | 173.3 | 176.4 |
| $GeR^1$ | 140.9 134.7 128.2 129.0 | 135.9 134.8 129.1 128.3 | 136.3 134.6 127.9 128.7 | 135.8 135.7 128.8 129.5 | 141.5 138.4 127.6 124.8 21.1 | 138.8 135.4 131.0 128.0 21.4 | 51.4 56.8 | 56.1 61.7 | 51.4 56.8 | 50.8 56.1 |
| $R^3$ | - | - | 18.4 | - | - | - | - | - | - | - |
| $R^2$ | 135.0 129.0 125.0 128.0 | - | - | 16.6 | 135.2 131.5 128.1 128.6 | 133.2 129.0 157.6 113.5 31.0 | 129.4 130.9 131.0 143.0 | 134.5 133.5 129.9 131.9 | 134.5 133.5 129.9 131.9 | 19.4 |
| $^{119}Sn$ | -117 | -484 | -544 | -123 | -189 | -165 | -118 | -312 | -128 | -189 |

For $^{13}C$ , $CDCl_3$ at 298 K(40%) ,$CDCl_3$ for $^{119}Sn$

The mechanisms involve in the bactericidal effect of these compounds have not been discerned. However, the site of action of the reference antibiotics is the cell wall. Bacteria used in this study were both gram negative and gram positive. It is well established that antibiotics that influence cell wall synthesis do not destroy gram negative bacteria. Thus, it would be of interest to investigate the effects of the dimethyltin dicarboxylates on both gram positive and gram negative bacteria. If there is a propensity of these compounds to effect the destruction of gram negative or antibiotic resistant bacteria that are unaffected by most antibiotics, then the possible use of these compounds in treating diseases caused by gram negative bacteria should be examined.

Table 5. Mass Fragmentation of selected compounds.

| Fragmentation | m/z | Fragmentation | m/z |
|---|---|---|---|
| $N(CH_2CH_2O)_3GeCHPhCH_2CO_2Sn^+Me_2$ | 518 | $(Ph_3GeCHPhCH_2CO_2)_2SnMe$ | 1041 |
| $PhCH{=}CHCOOSn^+Me_2$ | 297 | $Ph_3Ge^+$ | 305 |
| $N(CH_2CH_2O)_3GeCHPhCH_2CO_2^+$ | 368 | $Ph_2Ge^{+\cdot}$ | 228 |
| $PhCH{=}CHCOOSn^+$ | 267 | $PhCH{=}CHCOOSn^+$ | 267 |
| $N(CH_2CH_2O)_3Ge^+$ | 220 |  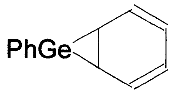 | 227 |
| $PhCH{=}CHCOO^+$ | 177 | $PhGe^+$ | 151 |
| $Me_2Sn^+$ | 150 | $Me_2Sn^+$ | 150 |
| $MeSn^{+\cdot}$ | 135 | $MeSn^{+\cdot}$ | 135 |
| $Sn^+$ | 120 | $HSn^{+\cdot}$ | 121 |
| $PhCH{=}CH^+$ | 103 | $PhCH{=}CH^+$ | 103 |

Table 6. Bactericidal data of some selected dimethyltin derivatives.

| **Name of Bacteria** | **Clinical Implication** | **Zone Inhibition (mm)** | | | | | **Ref. Drug** | |
|---|---|---|---|---|---|---|---|---|
| | | **I** | **II** | **III** | **IV** | **V** | **(a)** | **(b)** |
| *Bacillus cerus* | Food poisoning | 11.5 | 8.5 | 9 | 8.5 | 9.5 | 8 | 9 |
| *Corynebacterian dipheriae* | Diphtheria, infections of ear, nose, throat & skin | 11.5 | 9 | 9 | 7.5 | 9.5 | 16 | 14 |
| *E.coli* | Infection of wounds & urinary treat & dysentery | 10 | 7.5 | — | 7 | 7 | 10.5 | 10 |
| *Klebslella pneumoniae* | Septicemia, infection of respiratory tract | 10 | 7.5 | 6 | 7 | 6.5 | 8.0 | 8.5 |
| *Proteus mirabill* | Infection of urinary tract, septicemia | 10 | 8 | 7 | 7 | 9.5 | 11.0 | 11.5 |
| *Pseudomonas aeroginosa* | Infection of wounds, eyes, septicemia | — | 10 | 7.5 | — | — | 8.0 | 8.5 |
| *Salmonella typhi* | Typhoid fever, food poisoning, localized infection | — | 7 | — | — | — | 8.0 | 8.0 |
| *Shigella boydil* | Inflammation of GIT, bacterial dysentery | 7.5 | 9.5 | 7.5 | 6 | 6 | 18 | 19 |
| *Staphylococeus aureus* | Food poisoning, scaled skin syndrome, endocarditis | 7.5 | 7.5 | 7.5 | 7.5 | 7.5 | 14 | 16 |
| *Streptococeus pyogenes* | Actrile rheamatic fever, scarlet fever, septic sounds | 8 | 9.5 | 7.5 | 7.5 | — | †11 | 9 |

Key: – No activity.

† Decreased in bacterial population/unit area.

Colony forming unit (CFU) ml = $10^4$-$10^6$.

Size of well = 5mm (radius).

Ref. drug(a) = Amoxicillin $(H_2O)_3$.

Ref. drug(b) = Ampicillin $(H_2O)_3$

a=(*Invitro*) (agar well diffusion protocol) conc. 100μg/100μl of DMSO

**Acknowledgment**

MAC is thankful to the University of Azad Jammu & Kashmir, Muzaffarabad for the grant of a study leave. This research was supported in part by the National Institutes of Health Minority Biomedical Research Support Program, MBRS-SCORE (GM08005).

### References

1. S. J. Blunden and A. Chapman, in *Organometallic Compounds in the Environment*, P. J. Craig, (ed.) John Wiley & Sons, New York, 1986, p111.
2. J. S. Thayer, *Organometallic Compounds and Living Organism*, Academic Press, New York, 1984.
3. S. J. Blunden, P. A. Cusade and R. Hill, *The Industrial Uses of Tin Chemicals*, The Royal Society of Chemistry, London, 1985.
4. A. J. Crowe, P. J. Smith and G. Alassi, *Chem. Boil. Interactions,* **32**, 171 (1980).
5. (a) M. Gielen, P. Lilieveled, D. Devos and R. William, in *Metal Based Antitumour Drugs*, M. Gielen, (ed.), Friend Publishing House, Tel Aviv, Israel, 1992, p 29. (b) M. Gielen, *Coord. Chem. Rev.,* **151**, 41 (1996). (c) M. Kemmer, M. Gielen, M. Biesemans, D. de Vos and R. Willem, *Metal-Based Drugs* **5**, 189 (1998). (d) D. de Vos, R. Willem, M. Gielen, K. E. van Wingerden and K. Nooter, *Metal-Based Drugs* **5**, 179 (1998).
6. R. William, H. Dalil, P. Briekaert, M. Biesemans, L. Ghys, K. Norter, D. de Vos, F, Ribot and M. Gielen, *Main Group Met. Chem.,* **20**, 535 (1997).
7. H. Satoh and T. Iwagnchi, Antitumour Activity of a New Organogermanium Compound Ge-132, *Cancer Chemother*., **6**, 79 (1979).
8. N. Kakimoto, M. Matsui, T. Takada and M. Aluba, *Heterocycles,* **23**, 2681 (1985).
9. E. Lukevics, S. Germane and L. Ignatovich, *Appl. Organomet. Chem.*, **6**, 543 (1992).
10. E. Lukevics, P. Arsenyan and M. Viveries, *Metal Based Drugs,* **5**, 251 (1998).
11. S. Xueqing, Y. Zhiqiang, X. Qinglan and L. Jinshan, *J. Organomet. Chem.*, **566**, 103 (1998).
12. M. Danish, S. Ali, A. Badshah, M. Mazhar, H. Masood, A. Malik and G. Kehr, *Synth. Inorg. Met. Org. Chem.,* **26**, 863 (1997).
13. G. K. Sandhu and N.S. Boparoy, *J. Organomet. Chem.*, **89**, 411 (1991).
14. B. S. Manhas and A. K. Trikha, *J. Indian Chem. Soc.,* LIX, 315 (1982).
15. M. G. Voronkov and V. P. Baryshok, *J. Organomet. Chem.*, **239**, 199 (1982).
16. P. G. Harrison, in *Chemistry of Tin*, P. G. Harrison (ed.), Chapman and Hall, NY, 71, 1989.
17. T. P. Lockhart and W. F. Manders, *Inorg. Chem.,* **25**, 892 (1986).
18. A. Badshah, M. Danish, S. Ali, M. Mazhar, S. Mahmood and M. I. Chaudhary, *Synth. React. Inorg. Met.-Org.. Chem.,* **24**, 1155 (1994).
19. M. Danish, H. G. Alt, A. Badshah, S. Ali, M. Mazhar, and N. Islam, *J. Organomet. Chem.,* **486**, 51 (1995).
20. M. Danish, S. Ali, M. Mazhar, A. Badshah, M. I. Chaudhary, H. G. Alt, and G. Kehr, Polyhedron, **14**, 3115, (1995).
21. M. H. Bhatti, S. Ali, H. Masood, M. Mazhar and S. I. Qureshi, *Synth. React. Inorg. Met.-Org Chem.,* **30**, 1715 (2000).
22. B.Wrackmeyer, Annu. Rep. NMR Spectrosc., **38**, 203, (1999)
23. P. G. Harrison, in *Chemistry of Tin*, P. G. Harrison, (ed.) Chapmand and Hall, New York, NY, 1989, p76 and references therein.
24. S.Rozite, I. Mazeika, A. Gaukhman, N.P. Erchak, L.M. Ignatovich and E.Lukevics *J. Organomet. Chem.,* **384** 257 (1990)

**Received: January 1, 2002 – Accepted: January 19, 2002 –**

**Accepted in publishable format: March 19, 2002**

